# Change of Antibiotic Susceptibility Testing Guidelines from CLSI to EUCAST: Influence on Cumulative Hospital Antibiograms 

**DOI:** 10.1371/journal.pone.0079130

**Published:** 2013-11-01

**Authors:** Aline Wolfensberger, Hugo Sax, Rainer Weber, Reinhard Zbinden, Stefan P. Kuster, Michael Hombach

**Affiliations:** 1 Division of Infectious Diseases and Hospital Epidemiology, University Hospital and University of Zurich, Zurich, Switzerland; 2 Institute of Medical Microbiology, University of Zurich, Zurich, Switzerland; California Department of Public Health, United States of America

## Abstract

**Objective:**

We studied whether the change in antibiotic susceptibility testing (AST) guidelines from CLSI to EUCAST influenced cumulative antibiograms in a tertiary care hospital in Switzerland.

**Methods:**

Antibiotic susceptibilities of non-duplicate isolates collected within a one-year period before (period A) and after (period B) changing AST interpretation from CLSI 2009 to EUCAST 1.3 (2011) guidelines were analysed. In addition, period B isolates were reinterpreted according to the CLSI 2009, CLSI 2013 and EUCAST 3.1 (2013) guidelines.

**Results:**

The majority of species/drug combinations showed no differences in susceptibility rates comparing periods A and B. However, in some gram-negative bacilli, decreased susceptibility rates were observed when comparing CLSI 2009 with EUCAST 1.3 within period B: *Escherichia coli* / cefepime, 95.8% (CLSI 2009) vs. 93.1% (EUCAST 1.3), P=0.005; *Enterobacter cloacae* / cefepime, 97.0 (CLSI 2009) vs. 90.5% (EUCAST 1.3), P=0.012; *Pseudomonas aeruginosa* / meropenem, 88.1% (CLSI 2009) vs. 78.3% (EUCAST 1.3), *P*=0.002. These differences were still evident when comparing susceptibility rates according to the CLSI 2013 guideline with EUCAST 3.1 guideline. For *P. aeruginosa* and imipenem, a trend towards a lower antibiotic susceptibility rate in ICUs compared to general wards turned into a significant difference after the change to EUCAST: 87.9% vs. 79.8%, *P*=0.08 (CLSI 2009) and 86.3% vs. 76.8%, *P*=0.048 (EUCAST 1.3).

**Conclusions:**

The change of AST guidelines from CLSI to EUCAST led to a clinically relevant decrease of susceptibility rates in cumulative antibiograms for defined species/drug combinations, particularly in those with considerable differences in clinical susceptibility breakpoints between the two guidelines.

## Introduction

The European Committee for Antimicrobial Susceptibility Testing (EUCAST) was initiated to harmonize minimum inhibitory concentration (MIC) breakpoints across Europe [1]. In line with many European clinical laboratories, the University of Zurich’s Institute of Medical Microbiology, Switzerland, changed its antibiotic susceptibility testing (AST) system from the Clinical and Laboratory Standards Institute (CLSI) 2009 methodology to the EUCAST 1.3 AST guidelines on 1st July 2011 [2,3]. 

In general, EUCAST recommends lower resistance MIC breakpoints than CLSI, in particular for Gram-negative bacteria, and, in part, abandoned the intermediate susceptibility zone. These changes have been shown to result in different susceptibility rates, e.g. higher cefepime and meropenem resistance rates in *Pseudomonas aeruginosa* [4], higher ceftazidime and ceftriaxone resistance rates in *Escherichia coli* causing bacteremia [5], higher ceftazidime resistance in ESBL-producing *E. coli* and *Klebsiella pneumonia* [6], and higher cefepime and ceftazidime resistance in ESBL producing *E. coli* [7]. However, the actual effect of the guideline changes on cumulative hospital antibiograms is unknown, even though local cumulative antibiograms are important for guiding empirical antibiotic therapy [8,9], and changes in cumulative resistance rates may influence the choice of empirical antimicrobial treatment [10]. 

This study was designed to determine whether and to which extent susceptibility rates in cumulative antibiograms of the five most prevalent bacterial species in our tertiary-care hospital would differ between two consecutive years before and after changing from CLSI 2009 to EUCAST 1.3 (2011) AST guidelines. Furthermore, we determined whether differences in cumulative antibiograms represented true changes in antimicrobial susceptibility, or if they were merely an effect of guideline changes. 

In addition, as resistance rates of cumulative antibiograms from general ward specimens reportedly differ from those found on intensive care units (ICUs), we aimed to determine whether guideline dependent changes differed between ICUs and general wards [11,12].

## Materials and Methods

### Setting

The University Hospital Zurich, Zurich, Switzerland, is an 871 bed tertiary-care teaching hospital covering all medical specialties except paediatrics and orthopaedics. Six intensive care units (medical ICU, general, thoracic and transplant surgery ICU, trauma ICU, burn ICU, cardiac surgery ICU, neurosurgery ICU) with a total of 65 beds are assigned to different departments. Hematopoietic stem cell transplantations are performed in a specialized unit. All microbiologic samples are tested in the clinical microbiology laboratory of the Institute of Medical Microbiology, University of Zurich, Zurich, Switzerland.

### Data collection

Data were collected in two consecutive one-year periods just before (period A) and after (period B) the change of AST interpretation from CLSI 2009 to EUCAST 1.3 on 1 July 2011. All bacterial isolates from samples collected on general wards and intensive care units were taken into account. In addition, samples of the outpatients’ clinic for respiratory medicine were included in a subgroup analysis for *P. aeruginosa*. 

The species analysed comprised *E. coli*, *K. pneumoniae, Enterobacter cloacae*, *P. aeruginosa*, and *Staphylococcus aureus*. According to the guidelines for analysis and presentation of cumulative antibiograms, we excluded all repeated isolates, i.e. only the first isolate of a certain bacterium per patient and year was analysed, regardless of the material, the donor site, or the resistance profile [13]. 

In addition, isolates collected on the general wards and on the ICUs were analysed separately. The “ICU section” consisted of the six ICUs and the hematopoietic stem cell transplantation unit. The “ward-section” consisted of all general wards.

### Susceptibility testing

For susceptibility testing, the disc diffusion method according to Kirby-Bauer was used [14]. Antibiotic discs were obtained from i2a (Montpellier, France). Susceptibility testing was done on Mueller-Hinton agar (Becton-Dickinson, Franklin Lakes, NJ, USA) using MacFarland 0.5 from overnight cultures followed by incubation at 35°C for 16-18h. Inhibition zone diameters were determined and recorded in the automated Sirweb/Sirscan system (i2a) and interpreted according to CLSI 2009 and EUCAST 1.3 guidelines [2,3].

### Comparison of CLSI 2009 and EUCAST 1.3 (2011)

For certain drugs, e.g. ceftazidime, cefotaxime, and piperacillin/tazobactam, EUCAST guidelines contain other antibiotic disc loads than CLSI guidelines, preventing a direct comparison of disk diffusion AST results [2,3]. Thus, we only included drugs into analysis that have identical antibiotic disc loads in both CLSI and EUCAST guidelines, i.e., amoxicillin/clavulanic acid, cefuroxime, cefoxitin, ceftriaxone, cefepime, imipenem, meropenem, tobramycin, sulfomethoxazole/trimethoprim, ciprofloxacin, gentamicin, erythromycin, clindamycin, rifampin, and teicoplanin. 

The first one-year period (period A) was interpreted according to CLSI 2009 guidelines only. Period B was originally interpreted according to EUCAST 1.3 guidelines. In order to analyze whether or not changes in susceptibility rates over time were due to the guideline change alone, results of period B were reinterpreted according to CLSI 2009 guidelines. Additionally, period B was reinterpreted according to CLSI 2013 and EUCAST 3.1.

Isolates of intermediate susceptibility were classified together with resistant isolates to the “non-susceptible”-group. 

### Statistical analyses

Differences in group proportions were assessed using chi-square or Fisher’s exact tests, as appropriate. We used Stata (Version 12.1, StataCorp, College Station, Texas) for statistical analyses. *P*-values <0.05 were considered statistically significant.

## Results

### Cumulative hospital antibiogram

Between July 2010 and June 2011 (period A), 2540 isolates (1085 *E. coli*, 277 *K. pneumoniae*, 186 *E. cloacae*, 271 *P. aeruginosa*, 721 *S. aureus*) were included. Between July 2011 and June 2012 (period B), 2688 isolates (1177 *E. coli*, 310 *K. pneumoniae*, 200 *E. cloacae*, 282 *P. aeruginosa*, 719 *S. aureus*) were included.

**Figure 1:**
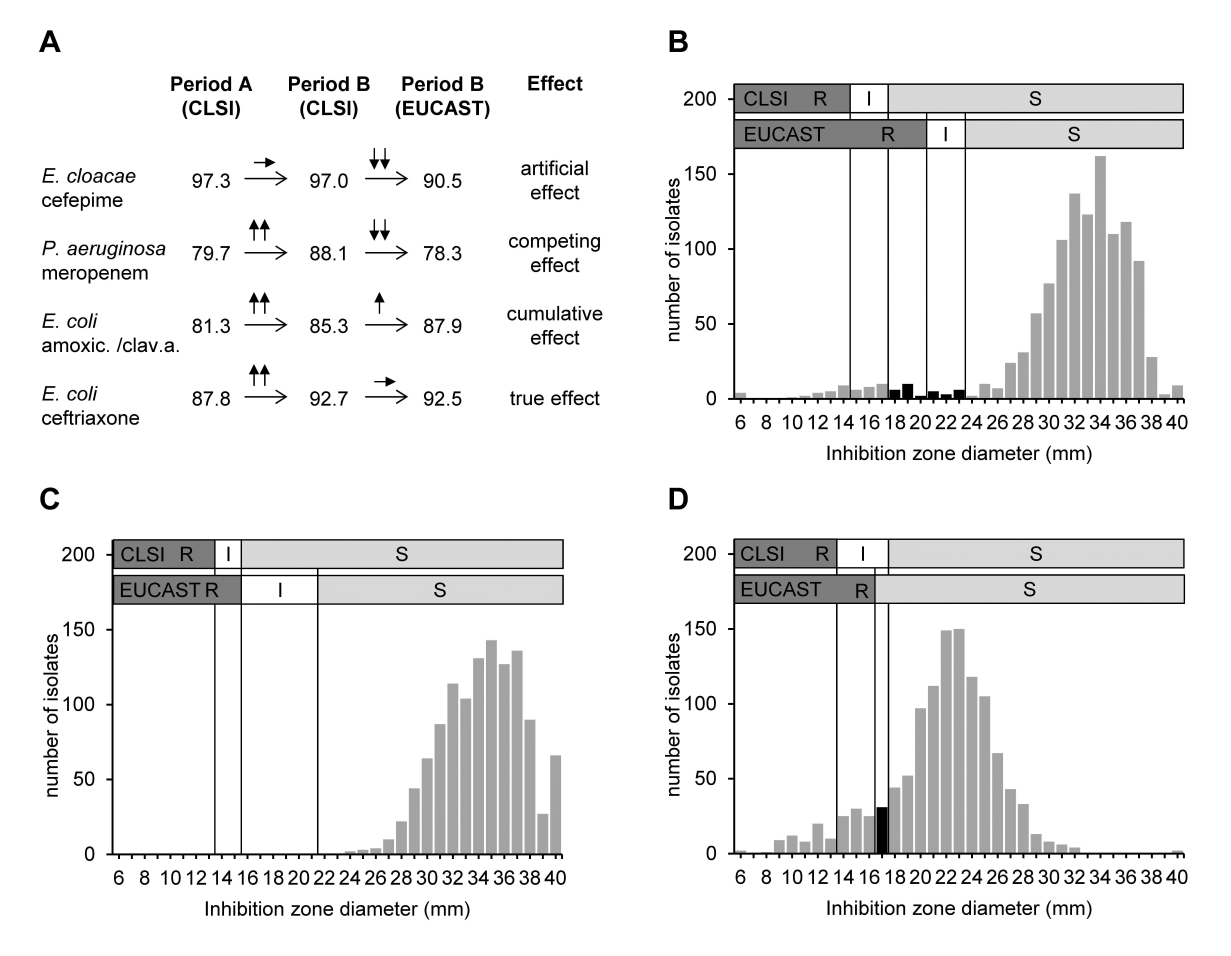
Different patterns of effects and distributions of inhibition zone diameters. Part A: Different “patterns of effects” when analysing period A according CLSI 2009 guidelines and period B according CLSI 2009 and EUCAST 1.3 guidelines; numbers are percent susceptible. Part B: Distribution of inhibition zone diameters of *E.coli* and cefepime: isolates of columns in black are not classified as “susceptible” any more when reported according to EUCAST 1.3 guidelines. Part C: Distribution of inhibition zone diameters of *E. coli* and meropenem: no change of susceptibility rate when EUCAST 1.3 guidelines are applied. Part D: Distribution of inhibition zone diameters of *E. coli* and amoxicillin/clavulanic acid: overlap of wild-type and resistent bacteria, leading to a change in classification from “intermediate” to “susceptible” (black column) of a near significant number of isolates when EUCAST 1.3 guidelines are applied.

Four different “patterns” of effects on susceptibility rates were found when comparing the cumulative antibiograms of period A and period B by either interpreting the antibiograms according to CLSI 2009 or according to EUCAST 1.3 AST guidelines (Figure 1). 

#### Pattern 1 - Artificial changes in susceptibility rates

A decrease in the cefepime susceptibility rates of *E. coli* and *E. cloacae* from period A to period B was observed (Table 1, comparison 1). These differences disappeared when periods A and B were both interpreted according to CLSI 2009 AST guidelines (Table 1, comparison 2). 

**Table 1:** Comparison of cumulative antibiograms of two adjacent one-year periods by either applying CLSI or EUCAST guidelines.

		**Comparison 1**			**Comparison 2**			**Comparison 3**			**Comparison 4**		
		**CLSI 2009**	**EUCAST 1.3 (2011)**		**CLSI 2009**	**CLSI 2009**		**CLSI 2009**	**EUCAST 1.3 (2011)**		**CLSI 2013**	**EUCAST 3.1 (2013)**	
Species	Drug	Period A^a^	Period B^b^	*P*- value	Period A^a^	Period B^b^	*P*- value	Period B ^b^	Period B^b^	*P*- value	Period B^b^	Period B^b^	*P*- value
*E.coli*	Amoxicillin/ clavulanic acid	81.3	87.9	<0.001	81.3	85.3	0.011	85.3	87.9	0.07	85.3	87.9	0.07
	Cefuroxime	89.6	90.9	0.32	89.6	90.9	0.32	90.9	90.9	1.00	90.9	90.9	1.00
	Ceftriaxone	87.8	92.5	0.001	87.8	92.7	<0.001	92.7	92.5	0.94	92.5	92.5	1.00
	Cefepime	97.1	93.1	<0.001	97.1	95.8	0.11	95.8	93.1	0.005	95.8	93.1	0.005
	Imipenem	100.0	100.0	n.a.	100.0	100.0	n.a.	100.0	100.0	n.a.	100.0	100.0	n.a.
	Meropenem	100.0	100.0	n.a.	100.0	100.0	n.a.	100.0	100.0	n.a.	100.0	100.0	n.a.
	Tobramycin	88.1	88.8	0.70	88.1	89.7	0.34	89.7	88.8	0.51	89.7	88.6	0.43
	Sulfomethoxazole/ trimethoprim	68.6	65.2	0.08	68.6	65.2	0.08	65.2	65.2	1.00	65.2	65.2	1.00
	Ciprofloxacin	80.0	81.3	0.46	80.0	81.2	0.49	81.2	81.3	0.96	81.2	81.3	0.96
*K. pneumoniae*	Amoxicillin/ clavulanic acid	85.6	92.3	0.011	85.6	89.7	0.13	89.7	92.3	0.33	89.7	92.3	0.33
	Cefuroxime	87.0	89.6	0.37	87.0	89.6	0.37	89.6	89.6	n.a.	89.6	89.6	1.00
	Ceftriaxone	88.6	91.6	0.28	88.6	91.6	0.28	91.6	91.6	1.00	91.6	91.6	1.00
	Cefepime	96.0	92.6	0.08	96.0	95.5	0.84	95.5	92.6	0.17	95.5	92.6	0.17
	Imipenem	98.9	99.4	0.67	98.9	99.4	0.67	99.4	99.4	1.00	98.4	99.0	0.72
	Meropenem	98.0	99.0	0.44	98.0	99.4	0.22	99.4	99.0	1.00	98.7	99.0	1.00
	Tobramycin	87.1	93.9	0.010	87.1	94.2	0.006	94.2	93.9	1.00	94.2	93.2	0.74
	Sulfomethoxazole/ trimethoprim	81.2	81.3	1.00	81.2	81.3	1	81.3	81.3	1.00	81.3	81.3	1.00
	Ciprofloxacin	88.8	93.5	0.06	88.8	93.9	0.037	93.9	93.5	1.00	93.9	93.5	1.00
*E. cloacae*	Amoxicillin/ clavulanic acid	4.8	9.0	0.12	4.8	7.5	0.30	7.5	9.0	0.72	7.5	9.0	0.72
	Cefuroxime	66.5	70.5	0.44	66.5	70.5	0.44	70.5	70.5	1.00	70.5	70.5	1.00
	Ceftriaxone	73.8	74.5	0.90	73.8	76.0	0.63	76.0	74.5	0.82	74.5	74.5	1.00
	Cefepime	97.3	90.5	0.006	97.3	97.0	1.00	97.0	90.5	0.012	97.0	90.5	0.012
	Imipenem	100.0	99.5	1.00	100.0	100.0	n.a.	100.0	99.5	1.00	95.0	98.5	0.09
	Meropenem	100.0	97.5	0.07	100.0	100.0	n.a.	100.0	97.5	0.06	97.0	97.5	0.77
	Tobramycin	95.7	96.0	1.00	95.7	96.0	1.00	96.0	96.0	1.00	96.0	96.0	1.00
	Sulfomethoxazole/ trimethoprim	93.0	91.0	0.57	93.0	91.0	0.57	91.0	91.0	1.00	91.0	91.0	1.00
	Ciprofloxacin	95.1	96.0	0.81	95.1	96.5	0.61	96.5	96.0	1.00	96.5	96.0	1.00
*P. aeruginosa*	Cefepime	85.6	88.7	0.31	85.6	88.7	0.31	88.7	88.7	1.00	88.7	88.7	1.00
	Imipenem	76.7	82.9	0.07	76.7	85.1	0.013	85.1	82.9	0.57	83.3	82.9	1.00
	Meropenem	79.7	78.3	0.74	79.7	88.1	0.013	88.1	78.3	0.002	86.3	78.3	0.015
	Tobramycin	91.6	94.6	0.21	91.6	94.6	0.21	94.6	94.6	1.00	94.6	94.6	1.00
	Ciprofloxacin	84.1	90.7	0.021	84.1	93.9	<0.001	93.9	90.7	0.20	93.9	90.7	0.20
*S. aureus*	Cefoxitin	96.7	94.4	0.041	96.7	94.4	0.041	94.4	94.4	1.00	94.4	94.4	1.00
	Gentamicin	98.2	98.7	0.52	98.2	98.9	0.38	98.9	98.7	1.00	98.9	98.7	1.00
	Sulfomethoxazole/ trimethoprim	99.2	99.3	1.00	99.2	99.3	1.00	99.3	99.3	1.00	99.3	99.3	1.00
	Erythromycin	87.2	87.8	0.74	87.2	86.9	0.87	86.9	87.8	0.63	86.9	87.8	0.63
	Clindamycin	97.6	98.4	0.33	97.6	98.4	0.33	98.4	98.4	1.00	98.4	98.4	1.00
	Rifampicin	99.4	98.6	0.18	99.4	98.7	0.27	98.7	98.6	1.00	98.7	98.6	1.00
	Teicoplanin	100.0	100.0	n.a.	100.0	100.0	n.a.	100.0	100.0	n.a.	100.0	100.0	n.a.

^a^1 July 2010 to 30 June 2011, numbers are percent susceptible

^b^1 July 2011 to 30 June 2012, numbers are percent susceptible

n.a. not applicable.

#### Pattern 2 - Competing effects

When comparing *P. aeruginosa* / meropenem susceptibility rates defined according to CLSI 2009 guidelines in both periods, more specimens were reported susceptible in period B than in period A (Table 1, comparison 2), reflecting a true epidemiological change. When period A was analysed according to CLSI 2009 and period B according to EUCAST 1.3 AST guidelines, the reported susceptibility rates did not differ (Table 1, comparison 1). A similar effect was shown for *P. aeruginosa* / imipenem and ciprofloxacin susceptibility rates.

#### Pattern 3 - Cumulative effects

Amoxicillin/clavulanic acid susceptibility rates of *E. coli* and *K. pneumonia* increased between period A and B when both periods were interpreted according to CLSI 2009 AST guidelines (Table 1, comparison 2). This increase was amplified by interpreting period B according to EUCAST 1.3 AST guidelines (Table 1, comparison 3).

#### Pattern 4 - True changes in susceptibility rates

*E. coli* ceftriaxone susceptibility and *K. pneumoniae* tobramycin susceptibility rates increased from period A to period B, while cefoxitin susceptibility rates of *S. aureus* decreased (Table 1, comparison 1). These changes in susceptibility rates remained when CLSI 2009 guidelines were applied to period B (Table 1, comparison 2). 

Table 1 shows that, besides changing AST patterns described above, there was no difference in susceptibility rates between the two periods in the majority of isolates, neither when comparing CLSI 2009 AST guidelines applied to period A and EUCAST 1.3 guidelines to period B (comparison 1), nor when periods A and B were both interpreted according to CLSI 2009 AST guidelines (comparison 2). The results of comparison 3 were unchanged when inhibition zone diameters of period B interpreted according to CLSI 2103 and EUCAST 3.1 were compared (comparison 4).

### Comparison of cumulative antibiograms of intensive care units, general wards and other units

Regardless of the methodology applied, susceptibility rates of *E. coli* to cefuroxime, ceftriaxone and cefepime, and susceptibility rates of *S. aureus* to clindamycin were lower in cumulative antibiograms of intensive care units as compared to those of general wards (Table 2). Similarly, a trend to lower susceptibility rates in ICUs could be detected in some other species/drug combinations.

**Table 2:** Comparison of cumulative antibiograms of wards vs. ICUs by either applying CLSI 2009 or EUCAST 1.3 (2011) guidelines to period B^a^.

		CLSI 2009				EUCAST 1.3 (2011)		
Species	Drug	Wards	ICUs	*P*-value		Wards	ICUs	*P*-value
*E. coli*	Amoxicillin/ clavulanic acid	85.9	82.4	0.20		88.4	85.7	0.29
	Cefuroxime	92.1	85.1	0.003		92.1	85.1	0.003
	Ceftriaxone	93.5	89.0	0.039		93.3	89.0	0.043
	Cefepime	96.6	92.4	0.012		94.2	88.2	0.004
	Imipenem	100	100	n.a.		100	100	n.a.
	Meropenem	100	100	n.a.		100	100	n.a.
	Tobramycin	90.5	86.2	0.08		89.5	85.2	0.07
	Sulfomethoxazole/ trimethoprim	65.0	65.9	0.87		65.0	65.9	0.87
	Ciprofloxacin	81.6	79.1	0.44		81.8	79.1	0.38
*K. pneumoniae*	Amoxicillin/clavulanic acid	89.9	89.3	0.84		92.5	91.7	0.81
	Cefuroxime	89.3	90.5	0.84		89.3	90.5	0.84
	Ceftriaxone	91.2	92.9	0.82		91.2	92.9	0.82
	Cefepime	95.1	96.4	0.77		92.5	92.9	1.00
	Imipenem	99.6	98.8	0.47		99.6	98.8	0.47
	Meropenem	99.6	98.8	0.47		99.1	98.8	1.00
	Tobramycin	94.7	92.9	0.59		94.2	92.9	0.60
	Sulfomethoxazole/ trimethoprim	79.6	85.7	0.25		79.6	85.7	0.25
	Ciprofloxacin	93.4	95.2	0.79		93.4	94.0	1.00
*E. cloacae*	Amoxicillin/clavulanic acid	7.6	7.4	1.00		9.8	7.4	0.79
	Cefuroxime	72.0	67.6	0.62		72.0	67.6	0.62
	Ceftriaxone	77.3	73.5	0.60		75.8	72.1	0.61
	Cefepime	96.2	98.5	0.67		90.9	89.7	0.80
	Imipenem	100	100	n.a.		99.2	100	1.00
	Meropenem	100	100	n.a.		97.0	98.5	0.66
	Tobramycin	94.7	98.5	0.27		94.7	98.5	0.27
	Sulfomethoxazole/ trimethoprim	88.6	95.6	0.12		88.6	95.6	0.12
	Ciprofloxacin	96.2	97.1	1.00		95.4	97.1	0.72
*P. aeruginosa*	Cefepime	88.0	89.9	0.70		88.0	89.9	0.70
	Imipenem	87.9	79.8	0.08		86.3	76.8	0.048
	Meropenem	90.5	83.8	0.12		80.8	73.7	0.18
	Tobramycin	95.0	93.9	0.78		95.0	93.9	0.78
	Ciprofloxacin	93.3	94.9	0.79		90.6	90.9	1.00
*S. aureus*	Cefoxitin	94.1	95.2	0.72		94.1	95.2	0.72
	Gentamicin	98.8	99.0	1.00		98.6	99.0	1.00
	Sulfomethoxazole/ trimethoprim	99.0	100	0.33		99.0	100	0.33
	Erythromycin	86.4	87.9	0.63		87.5	88.4	0.80
	Clindamycin	99.2	96.6	0.02		99.2	96.6	0.02
	Rifampicin	98.6	99.0	1.00		98.4	99.0	0.73
	Teicoplanin	100	100	n.a.		100	100	n.a.

^a^1 July 2011 to 30 June 2012, numbers are percent susceptible

n.a. not applicable.

For *P. aeruginosa* and imipenem, a trend towards a lower antibiotic susceptibility rate in ICUs compared to general wards turned into a significant difference after the change to EUCAST 1.3 (Table 2). Moreover, in *P. aeruginosa* isolates collected in the outpatients’ clinic for respiratory medicine, which include specimens of numerous patients with cystic fibrosis and lung transplant recipients, a decrease in ciprofloxacin susceptibility rate was notable when applying EUCAST 1.3, but not CLSI 2009 guidelines (data not shown).

## Discussion

This observational study was designed to analyse cumulative hospital antibiograms in two adjacent one-year periods before and after the clinical laboratory changed antibiotic susceptibility test interpretation from CLSI 2009 to EUCAST 1.3 (2011) guidelines, and to analyse whether possible differences are due to true epidemiologic changes or result only from CLSI / EUCAST guideline differences. Differences resulting from guideline changes alone may misdirect physicians in the interpretation of antibiotic susceptibility trends in that truly increasing resistance rates may be missed or unchanging resistance rates may be reported as increasing or decreasing, resulting in a change of antibiotic use patterns and thus patient management and quality of care. 

Clinical breakpoint (CBP) setting is a multi-step process comprising the determination of epidemiological cut off (ECOFF) values from MIC distributions, correlating these ECOFFs to zone diameters distributions, comparing putative CBPs to available PK/PD data and, finally, clinical validation of putative CBPs in clinical outcome studies [15]. 

CLSI and EUCAST use different methods for the determination of disc diffusion CBPs: CLSI uses a variant of the error-rate-bounded method, sometimes incorporating an intermediate zone [16], and EUCAST first defines MIC breakpoints on the basis of epidemiological MIC cut-offs (ECOFFs) and pharmacokinetic/pharmacodynamic (PK/PD) parameters, and correlates those MIC CBPs to zone diameter values using the “MIC-coloured zone diameter histogram technique” [17,18]. The EUCAST policy of CBP setting promises more transparency in this still complex, rather consensus based process as all documents on diameter/MIC distributions, and ECOFF data are publicly available [19]. 

Such different CBP determination methods inevitably lead to different CBPs in CLSI and EUCAST AST guidelines for many species/drug combinations. EUCAST 1.3 (generally unchanged in EUCAST 2.0 and 3.1) disk diffusion CBPs are frequently higher as compared to CLSI 2009, and in many cases even higher as compared to revised CLSI guideline versions 2010 to 2013 [2,3,20-24]. Several studies have shown a significant impact of guideline changes on the reporting of AST results [4-7].

Instead of a uniform trend towards lower reported susceptibility rates for all species/drug combinations after implementation of EUCAST guidelines, this study showed four distinct patterns of effects of guidelines changes on susceptibility rates: i) a clearly artificial change of susceptibility rates due to changes in AST guidelines (e.g. *E. coli* / cefepime and *E. cloacae* / cefepime; Table 1, comparison 3); ii) competing effects of artificial changes in susceptibility rates and true epidemiologic variations (e.g. *P. aeruginosa* / meropenem; Table 1, comparisons 2 and 3); iii) cumulative effects resulting from artificial changes enhancing a true epidemiologic variation (e.g. *E. coli*/amoxicillin and clavulanic acid; Table 1, comparisons 2 and 3) and iv) a true change of susceptibility rates due to epidemiologic variation (e.g. *E. coli* / ceftriaxone; Table 1, comparison 2).

Three species/drug combinations (*E. coli* / cefepime, *E. cloacae* / cefepime and *P. aeruginosa* / meropenem) showed a statistically significant, yet artificial, decrease in susceptibility rates when period B was analysed according to EUCAST 1.3 (2011) instead of CLSI 2009 guidelines. For these three species/drug combinations, susceptible clinical inhibition zone diameter breakpoints differ substantially between EUCAST 1.3 and CLSI 2009 guidelines, i.e., 6 mm difference for cefepime susceptible CBP and *Enterobacteriaceae* (susceptible CPB CLSI 2009 18 mm; EUCAST 1.3 24 mm), and 8 mm difference in meropenem CBP for *P. aeruginosa* (susceptible CPB CLSI 2009 16 mm; EUCAST 1.3 24 mm). 

The present study showed significant changes in susceptibility rates for individual species/drug combinations with substantial differences in susceptible CBPs between both former and current CLSI and EUCAST guidelines. The probability and extent of changes, however, both depend on the inhibition zone diameters distributions of individual species/drug combinations and, thus, depend on the epidemiological situation present. This is seen in *E. coli* and meropenem, where a 6 mm difference between CBPs does not lead to changes in susceptibility rates (Figure 1b and c). 

Most important, some species/drug combinations, for which wild-type and resistant population zone diameters are not clearly separated, may be affected by only minor CBP changes, e.g. *E. coli* and amoxicillin/clavulanic acid (Figure 1d), for which a susceptible CBP difference of 1 mm (susceptible CBP CLSI 2009 18 mm; EUCAST 1.3 17 mm) led to an almost significant increase in susceptibility rates from 85.3% to 87.9% (Table 1, Comparison 3). 

Empirical antibiotic treatment is not only guided by individual patient characteristics, but also by epidemiological data such as local susceptibility rates [9]. Usually, empirical antibiotic treatment of specific pathogens or specific infections is recommended only if a certain level of resistance is not exceeded. Examples are the empirical treatment of cystitis with a threshold of 80% susceptible isolates for any antibiotic agent [25], or community acquired pneumonia and macrolide-therapy with a threshold of 75% susceptible isolates [26]. Consequently, changes in reported susceptibility rates will influence empirical antibiotic therapy. Meropenem susceptibility of *P. aeruginosa* dropped from 88.1% to 78.3% when analyzed according to CLSI 2009 and EUCAST 1.3 guidelines. This significant decrease in the meropenem susceptibility rate by EUCAST interpretation may, therefore, have a practical impact on the choice of the empirical antibiotic therapy in patients with life-threatening infections, and could result in more toxic (e.g. aminoglycosides, colistin) or less effective antibiotic regimens. 

The results of this study are in concordance with those of other authors demonstrating that susceptibility rates differ between ICU’s and general wards [11,27]. Corresponding differences were observed for cephalosporin susceptibility rates of *E. coli*, and clindamycin susceptibility rates of *S. aureus* when applying both CLSI and EUCAST guidelines. However, for imipenem susceptibility rates of *P. aeruginosa*, a significant difference between ICU’s and general wards was found only if applying EUCAST 2011, but not with CLSI 2009 (Table 2). Moreover, ciprofloxacin susceptibility rates of *P.aeruginosa* isolates collected in the outpatient clinic for respiratory medicine decreased significantly when EUCAST 2011 guidelines were applied. The most likely reason is a different inhibition zone diameter distribution (i.e. a shift towards lower inhibition zone diameters) of isolates originating from this patient population, resulting in a comparably stronger effect of guideline changes on susceptibility rates. These two examples show that guideline changes can affect different hospital wards to a different extent. 

Thus, effects of AST guidelines are not easily predictable and are dependent on the epidemiological situation. Both clinicians and microbiologists should, therefore, know their local epidemiology to be able to foresee effects of AST guideline changes on clinical practice. In addition, switching guidelines should be accompanied by thorough analyses during one time period where both guidelines are applied in order to detect bacteria/drug combinations belonging to one of the patterns described here. This might be particularly important in settings with higher resistance rates.

Despite a broad general set of data, the smaller sample size for the general ward/ICU distinction may have obscured significant differences in this study. In addition, this study is limited to the epidemiological situation in one single institution in north-eastern Switzerland, which is a low prevalence region for antibiotic resistance, hampering generalizability to other geographic settings. Effects of CBP changes as shown in this work may, thus, differ from the situation in high-prevalence regions. 

In conclusion, this study demonstrates that changes in AST guidelines, e.g. from CLSI 2009 to EUCAST 1.3 (2011) guidelines, can influence a hospital’s cumulative antibiogram in various ways. These changes cannot be easily predicted and may differ between hospital units. Even cautious interpretation can only serve as an approximation of the real epidemiological changes. Nevertheless, these issues have to be taken into account when interpreting cumulative antibiograms in a period after significant AST guideline changes. Further studies are needed to assess the effect of the guideline changes on different local epidemiological situations. 
